# Nanoscale mapping of quasiparticle band alignment

**DOI:** 10.1038/s41467-019-11253-2

**Published:** 2019-07-23

**Authors:** Søren Ulstrup, Cristina E. Giusca, Jill A. Miwa, Charlotte E. Sanders, Alex Browning, Pavel Dudin, Cephise Cacho, Olga Kazakova, D. Kurt Gaskill, Rachael L. Myers-Ward, Tianyi Zhang, Mauricio Terrones, Philip Hofmann

**Affiliations:** 1 Department of Physics and Astronomy, Interdisciplinary Nanoscience Center, Aarhus University, 8000 Aarhus C, Denmark; 2 National Physical Laboratory, Hampton Road, Teddington, TW11 0LW UK; 3 Central Laser Facility, STFC Rutherford Appleton Laboratory, Didcot, OX11 0QX, UK; 4 Diamond Light Source, Division of Science, Didcot, OX11 0DE, UK; 5 U.S. Naval Research Laboratory, Washington, DC 20375 USA; 6 Department of Physics and Center for 2-Dimensional and Layered Materials, Department of Materials Science and Engineering, The Pennsylvania State University, University Park, PA 16802 USA

**Keywords:** Nanoscale devices, Condensed-matter physics

## Abstract

Control of atomic-scale interfaces between materials with distinct electronic structures is crucial for the design and fabrication of most electronic devices. In the case of two-dimensional materials, disparate electronic structures can be realized even within a single uniform sheet, merely by locally applying different vertical gate voltages. Here, we utilize the inherently nano-structured single layer and bilayer graphene on silicon carbide to investigate lateral electronic structure variations in an adjacent single layer of tungsten disulfide (WS_2_). The electronic band alignments are mapped in energy and momentum space using angle-resolved photoemission with a spatial resolution on the order of 500 nm (nanoARPES). We find that the WS_2_ band offsets track the work function of the underlying single layer and bilayer graphene, and we relate such changes to observed lateral patterns of exciton and trion luminescence from WS_2_.

## Abstract (web summary)

Sharp atomic interfaces between materials dictate the interface’s electronic properties. The authors use angle-resolved photoemission spectroscopy with a spatial resolution of ~500 nm to investigate the nanoscale electronic band structure and band alignment in a lateral heterostructure composed of WS_2_ placed on alternating nano-stripes of monolayer and bilayer graphene.

## Introduction

The construction of a two-dimensional (2D) electronic device, such as a *pn*-junction, can be envisioned using two strategies: The first is to smoothly join two 2D materials with different electronic properties, essentially following the established recipe for three-dimensional (3D) semiconductors. Alternatively, one can create junctions using a single uniform sheet of material placed over a suitably pre-patterned substrate^1–3^, exploiting the sensitivity of 2D materials to their environment via band alignment^4,5^, screening^6–9^, or hybridization^10–12^. This approach has several advantages, such as technical simplicity and the absence of a possibly defective interface^13,14^. However, the interaction between a 2D material and substrate is highly non-trivial and hitherto poorly understood: Even in the absence of hybridization or charge transfer, substrate-screening can lead to an asymmetric band gap change, creating a type II heterojunction within a single sheet of 2D material^1^. This environmental screening may even be employed to engineer the photoluminescence (PL) from excitons at the ${\bar{\mathrm{K}}}$ valley of single layer (SL) semiconducting transition metal dichalcogenides (TMDs)^15–17^, as demonstrated by placing a SL TMD on a variable number of graphene layers^18^ or on conventional metals and insulators^19^. Moreover, strong many-body effects lead to a complex connection between the quasiparticle band structure and the optical properties. On one hand, even strong changes of the quasiparticle band structure might only have a very minor influence on the optical band gap, due to the interplay of the quasiparticle band gap size and exciton binding energy^6^. On the other hand, the quasiparticle band structure can greatly affect the formation of more complex entities such as trions^20^.

**Fig. 1:**
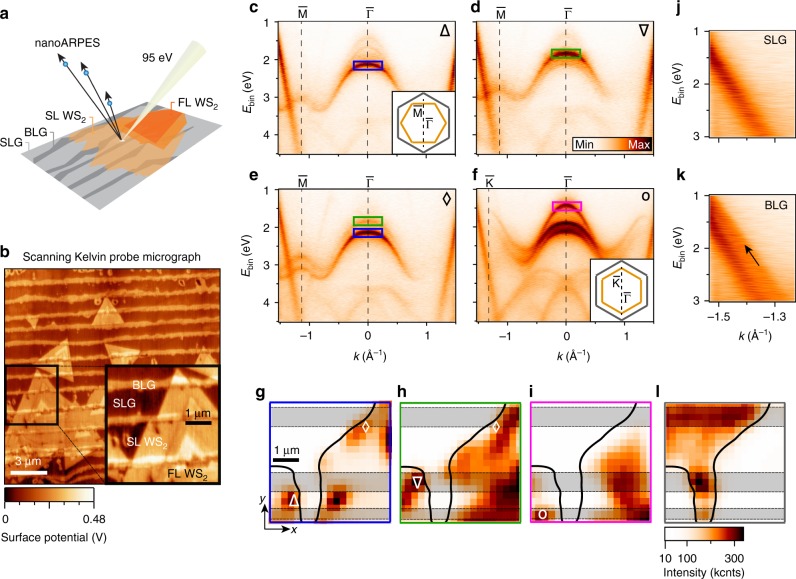
Nanoscale spatial mapping of WS_2_ electronic dispersion on a variable number of graphene layers. **a** Sketch of the nanoARPES experiment. **b** Scanning Kelvin probe micrograph showing the variation in surface potential over a typical area of the sample. The inset highlights an area containing SLG and BLG, as well as SL and FL WS_2_, similar to the samples measured with nanoARPES. **c**–**f** Representative ARPES spectra binned over 500 × 500 nm^2^ areas in the real space maps of the photoemission intensity in panels (**g**–**i**). The symbols Δ, ∇, ♢, ο on the maps in (**g**–**i**) indicate where a given (*E*, *k*)-dispersion in the correspondingly labeled panel in (**c**–**f**) was extracted. The Brillouin zones in the insets of (**c**, **f**) (gray for graphene, orange for WS_2_) give the orientation of the WS_2_ island in the acquisition area with respect to the underlying graphene for panels (**c**–**e**) and (**f**), respectively, along with the cut direction for the measurement (black dashed line). High symmetry points refer to the WS_2_ Brillouin zone. All data have been acquired along the ${\bar{\mathrm{K}}}$–${\bar{\mathrm{\Gamma }}}$–${\bar{\mathrm{K}}}\prime$ high symmetry direction of the single-domain graphene Brillouin zone as explained in Supplementary Note 1 and Fig. S1. The intensity maps in (**g**–**i**) are composed from the photoemission intensity in the (*E*, *k*)-regions marked with boxes of the same color in (**c**–**f**). An outline of the WS_2_ island edges has been drawn and the underlying BLG stripes are highlighted by gray-shaded boxes. **j**, **k** Detailed dispersion around the graphene branches from **j** SLG and **k** BLG stripes. **l** Intensity map obtained from the BLG branch marked by an arrow in (**k**). The color scale bar in (**l**) also applies to (**g**–**i**)

Here, we investigate the interplay of quasiparticle band alignments and optical properties in a lateral heterostructure of semiconducting SL WS_2_ placed on alternating areas of SL graphene (SLG) and bilayer graphene (BLG) grown on SiC. Since BLG has a tendency to nucleate at the step edges of SiC, we are able to study how the electronic structure and light–matter interaction varies on the nanoscale, due to the lateral change of the work function between SLG and BLG areas on SiC^21,22^. This demonstrates the possibility to utilize a specific substrate pattern to control the optoelectronic properties of an adjacent TMD. We directly visualize how the electronic structure changes at the complex heterogeneous atomic-scale interfaces present in our samples using nanoARPES; see illustration in Fig. 1a. This groundbreaking technique for electronic structure characterization provides three key new insights for the type of van der Waals heterostructure investigated here, which could not be accessed in conventional ARPES measurements that merely reveal the laterally averaged electronic structure (for example, in TMDs synthesized on metal substrates^23,24^ or graphene/SiC substrates^25–27^): (*i*) We can determine the energy- and momentum-dependence of band alignments at truly 2D interfaces, (*ii*) we obtain detailed spatially resolved information on how the electronic structure of a 2D semiconductor is modified around the one-dimensional (1D) SLG/BLG interface, and (*iii*) we can spatially disentangle the electronic dispersions of SL WS_2_ and few-layer (FL) WS_2_, and distinguish between islands of different orientations.

## Results

### Mapping of surface potential and electronic structure

Figure 1b shows the morphology and microscopic surface potential of WS_2_ islands on graphene measured by scanning Kelvin probe microscopy (SKPM) under ambient conditions. Triangular WS_2_ islands are observed with SL regions near the edges and FL areas towards the center. Alternating stripes of BLG and SLG are visible in both bare and WS_2_-covered areas. The strong contrast difference between WS_2_ placed on alternating stripes of BLG and SLG is caused by the large work function difference on the order of 100 meV^28^. The SL WS_2_ islands have a negligible influence on the relative work function difference between the underlying SLG and BLG, as confirmed by density functional theory calculations^28^.

Figure 1c–f presents the (*E*, *k*)-dependence of the topmost WS_2_ valence bands (VBs) measured from (500 × 500) nm^2^ areas on the sample using nanoARPES, extracted at the locations indicated with corresponding markers on the real space maps in Fig. 1g–i (for a large-scale overview see Fig. S2 in the Supplementary Material). Typically, a sharp and intense state is observed at ${\bar{\mathrm{\Gamma }}}$ that can be assigned to the local VB maximum (VBM) of SL WS_2_^29,30^. Upon close inspection, the binding energy of the VBM turns out to depend on the position within a WS_2_ island. In most cases, the VBM is found at either the energy shown in Fig. 1c or that in Fig. 1d. These two different energy regions have thus been marked by a blue and green box, respectively. In some areas, it is even possible to observe the simultaneous presence of two rigidly shifted SL WS_2_ VBs (Fig. 1e). The dispersion in Fig. 1f, on the other hand, is strikingly different from the other examples, showing a three-fold splitting with nearly equal intensity distribution between the split bands at ${\bar{\mathrm{\Gamma }}}$. The WS_2_ islands tend to orient either with the ${\bar{\mathrm{\Gamma }}}$–${\bar{\mathrm{M}}}$ (see Fig. 1c–e) or the ${\bar{\mathrm{\Gamma }}}$–${\bar{\mathrm{K}}}$ (see Fig. 1f) high symmetry directions aligned with the underlying graphene, although we occasionally find other orientations.

Further insight into local variations in the dispersion is obtained by investigating the spatial intensity distribution of the split states at ${\bar{\mathrm{\Gamma }}}$, as shown in Fig. 1g–i. These images correspond to real space maps of the photoemission intensity composed from the (*E*, *k*)-regions demarcated by boxes of the same color in Fig. 1c–f. The maps have been measured in scanning steps of 250 nm over a (4.5 × 4.5) μm^2^ area, thereby covering the edges of two adjacent WS_2_ islands of different orientations, as in the very similar region imaged by SKPM in the inset of Fig. 1b. The two SL WS_2_ VBs at different binding energy positions originate from distinct areas close to the edges where they give rise to the intense spots in Fig. 1g, h. The topmost split VB states (see magenta box in Fig. 1f) are concentrated towards the interior of the WS_2_ islands, where mainly FL structures occur, as evidenced by SKPM in Fig. 1b. In fact, the band structure in Fig. 1f is easily identified as being caused by multilayer splitting rather than simple shifts due to the visibly different effective mass (inverse curvature) of the topmost band.

We show that the shift between the VBs in Fig. 1c–e is correlated with the thickness of the underlying graphene by composing a real space map from the photoemission intensity of a BLG band. BLG is characterized by a splitting of the linear π-band near the ${\bar{\mathrm{K}}}_{}^{}$ point as shown in Fig. 1j, k (see arrow in panel (k) for the second branch). Mapping the intensity from this second branch permits a straightforward identification of BLG stripes in Fig. 1l; and this has been used to mark the gray-shaded boxes in all the real space maps. The BLG stripes are found to coincide with areas where the SL WS_2_ VB is shifted to lower binding energies, see Fig. 1d, h. Additional details of the correlation between graphene thickness and SL WS_2_ VB binding energy positions are discussed in Supplementary Notes 3 and 4 and Figs. S3 and S4.

**Fig. 2:**
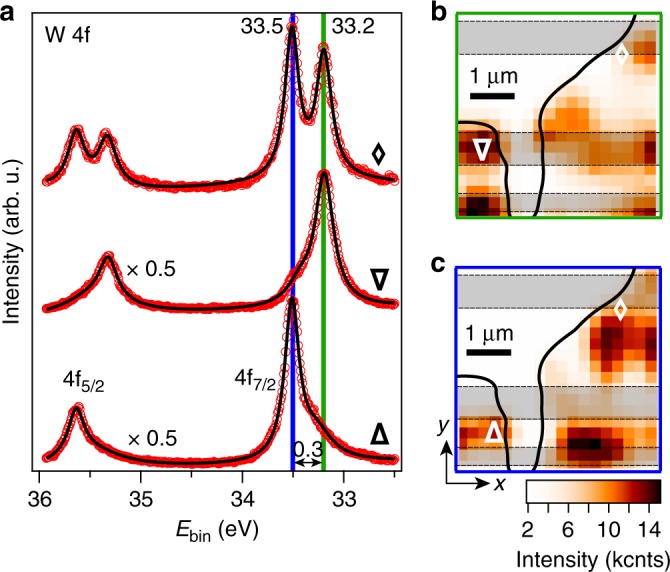
Spatial distribution of W 4*f* core level intensity. **a** Core levels from the same areas as the band structures in Fig. 1c–e, labeled by corresponding symbols. The data (open red circles) have been fitted with Lorentzian peaks (black curves). The resulting peak positions are stated in units of eV and marked by vertical blue and green bars. A separation of 0.3 eV between two shifted core levels is shown via a double-headed arrow. Note the intensity scaling factor of 0.5 for two of the curves. **b**, **c** Spatial distribution of photoemission intensity from the binding energies marked by vertical **b** green and **c** blue bars in (**a**). Symbols mark the areas that the core levels in (**a**) derive from. Gray-shaded boxes outline the BLG regions as in Fig. 1g–l

The nanoARPES data from the VB can be complemented by angle-integrated core level spectra, in the expectation that the core level binding energy should track an offset in the VB alignment between different areas, at least in a simple single-particle picture. Figure 2 presents nanoscale W 4*f* core level measurements collected over the same area as the VB spectra used to construct Fig. 1c–e. Each of the spin–orbit split components consists of two peaks separated by 0.3 eV. By plotting the spatial distribution of the photoemission intensity at each of the peak energies marked by green and blue bars, we obtain the maps shown in Fig. 2b, c corresponding to the VB analysis in Fig. 1. The peak at a lower binding energy (see green bar in panel (a)) appears to coincide with BLG areas as seen in panel (b) while the peak at a higher binding energy (see blue bar in panel (a)) is concentrated on the SLG areas as seen in panel (c). The trend is thus consistent with the spatial VB maps in Fig. 1g, h.

### Exciton and trion luminescence

**Fig. 3:**
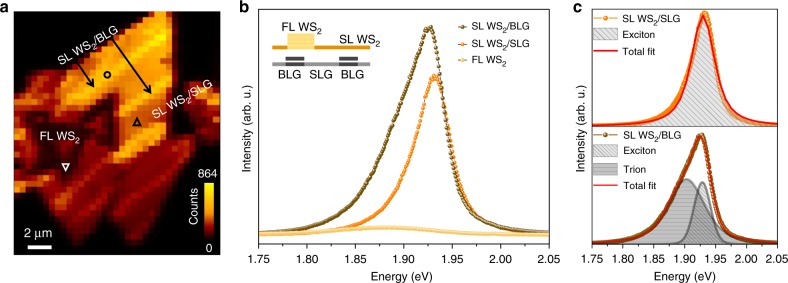
Spatial distribution of PL intensity and associated spectral response. **a** PL intensity map of representative WS_2_ island showing alternating regions of enhanced intensity and quenched signal for thick area at the centre of the island. The enhanced PL coincides with areas where WS_2_ overlaps with BLG stripes, with some examples (yellow stripes) indicated by black arrows. The wider areas of lower PL intensity correspond to SL WS_2_ on SLG. **b** Comparison of photoluminescence spectra associated with SL WS_2_ on SLG, SL WS_2_ on BLG and with FL WS_2_. These are extracted from the locations indicated by symbols in (**a**). **c** PL peak deconvolution, carried out using Lorentzian shape components, for SL WS_2_ on SLG (top panel) and SL WS_2_ on BLG, highlighting the appearance of the trion (bottom panel)

We turn to the consequences of this spatially heterogeneous electronic structure for the luminescence of excitons and trions in WS_2_^31^. PL mapping of a WS_2_ island, acquired under ambient conditions, is shown in Fig. 3a, where a stronger PL signal is observed on SL WS_2_ on BLG compared to SL WS_2_ on SLG. The energies of characteristic lines associated with SL WS_2_ on SLG and on BLG are identified in the PL spectra displayed in Fig. 3b. Detailed analysis by curve fitting to Lorentzian line shapes in Fig. 3c reveals an additional component for SL WS_2_ on BLG (bottom panel), attributed to charged exciton states (trions) at an energy of 1.90 eV, whereas the neutral exciton peak is found at 1.93 eV for both WS_2_ on SLG (top panel) and BLG. The position of the neutral exciton peak is shifted by ≈100 meV compared to SiO_2_ supported heterostructures of WS_2_ and graphene^18^, which may be explained by a change of doping (and thus screening) of the graphene on our SiC substrate. We note that patterns of exciton and trion luminescence have been observed in TMD flakes previously^32,33^ and interpreted in terms of a change in the chemical composition between the transition metal and chalcogen atoms^22^. Finally, Fig. 3a, b shows a weak PL response from the island’s centre, which is ascribed to the presence of FL WS_2_ and the indirect band gap of this material.

### Determination of band offsets

**Fig. 4:**
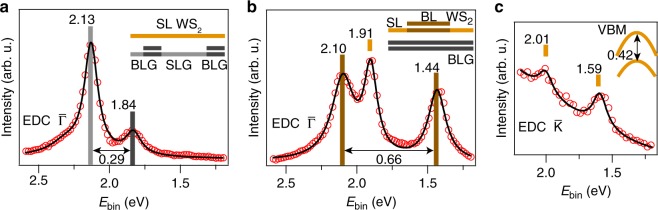
Analysis of band alignments. **a**, **b** Energy distribution curves (EDCs, red circles) binned within ±0.1 Å^−1^ around the ${\bar{\mathrm{\Gamma }}}$-point in the dispersions in Fig. 1e, f, respectively. The analysis is carried out **a** on a SL WS_2_ island straddling SLG and BLG patches, and **b** on mixed SL and bilayer WS_2_ supported on BLG (see side views in the insets). Peak positions obtained from fits to Lorentzian line shapes (black curves) on a constant background are given in units of electron volts. The tick marks above the peaks have been colored according to the structural diagrams and the spatial region where the peak originates from. **c** Similar EDC analysis as in (**a**, **b**) but carried out at the ${\bar{\mathrm{K}}}$-point in the dispersion in Fig. 1f. The fitted peak positions provide the VBM and spin–orbit splitting (see inset)

In order to obtain more accurate values for the band offsets, we analyze energy distribution curve (EDC) cuts at ${\bar{\mathrm{\Gamma }}}$ for the different structures. Figure 4a presents an EDC from the spectrum in Fig. 1e where a SL WS_2_ island straddles SLG and BLG stripes (see inset in Fig. 4a). Curve fitting of the peak positions reveals a binding energy shift of the WS_2_ of 0.29(5) eV between SLG and BLG, which matches the separation of the core level peaks in Fig. 2a. Performing a similar EDC analysis of the spectrum in Fig. 1f reveals that a splitting of 0.66(1) eV occurs between the states at lowest and highest binding energies, which matches the expected splitting of bilayer WS_2_^34^. The additional peak at 1.91(2) eV between the bilayer WS_2_ bands is attributed to a SL region (see inset in Fig. 4b) on a BLG stripe. We observe binding energy variations of up to 70 meV between VB peak positions in SL WS_2_ on the same substrate regions, which is evident from the two different binding energies in SL WS_2_ on BLG in Fig. 4a, b. However, we did not find a systematic trend in these small binding energy shifts. We speculate that details in the chemical composition within each flake may give rise to shifts on this energy scale as demonstrated for WS_2_ synthesized on titania^22^.

The data in Fig. 1f also provides access to the ${\bar{\mathrm{K}}}$ point of WS_2_, which is characterized by spin–orbit split bands that form the global VBM in SL WS_2_. ${\bar{\mathrm{K}}}$ is not accessible in the other spectra in Fig. 1c–e because of the rotated Brillouin zone. The EDC fit in Fig. 4c yields a spin–orbit splitting of 0.42(4) eV, in agreement with previous studies of SL WS_2_ in van der Waals heterostructures^20,30^, and a VBM of 1.59(4) eV for SL WS_2_ on BLG. By rigidly correcting for the shift on SLG areas one would thus expect the VBM on those regions around a binding energy of 1.9 eV. Under the assumption that the direct quasiparticle band gap of SL WS_2_ on SLG and BLG is smaller than 2.4 eV measured on silica^16^, we can infer that our WS_2_ remains *n*-type doped in the entire sample, although the density of free electrons will be substantially higher in SL WS_2_ on SLG.

## Discussion

**Fig. 5:**
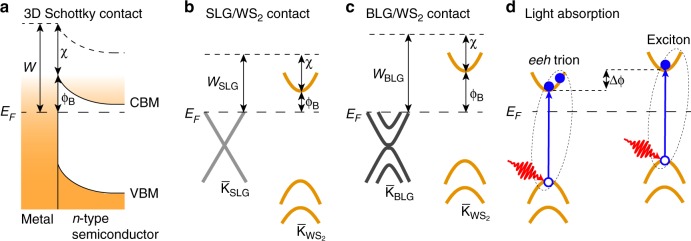
Interpretation of band alignments and luminescence features. **a** Typical band diagram for a 3D Schottky contact between a metal and an *n*-type semiconductor. The double-headed arrows illustrate the alignments of work function *W*, Schottky barrier height *ϕ*_*B*_ and electron affinity *χ*. **b**, **c** 2D Schottky alignment diagrams for **b** a SLG/WS_2_ contact and **c** a BLG/WS_2_ contact in UHV. The trend of the band alignments has been derived from the EDCs in Fig. 4. **d** Sketch of optically induced trion (electron–electron–hole (eeh) type) and exciton processes in SL WS_2_ under a different bias Δ*ϕ* caused by the varying Schottky barrier heights. Full (hollow) spheres correspond to electrons (holes), the red arrow corresponds to an optical pulse and the blue arrow signifies an electron–hole pair excitation

We have now tracked both the band offsets, W 4*f* core level energies and the excitonic spectrum across the SLG–BLG interface beneath SL WS_2_ and are thus in a position to explore the connection between these. The rigid VB and core level shifts of WS_2_ on SLG and BLG areas are consistent with an ideal 2D Schottky contact between WS_2_ and graphene. In order to see this, consider first a sketch of the band alignments for 3D metal–semiconductor junctions in Fig. 5a. The Schottky barrier height *ϕ*_*B*_ is set by the metal work function *W* and semiconductor electron affinity *χ*, i.e., *ϕ*_*B*_ = *W* − *χ*. Forming a metal–semiconductor contact leads to band bending with a depletion region towards the bulk of the semiconductor. For the interface between two 2D materials, this is irrelevant and the band offset is expected to follow the sketch in Fig. 5b, c for WS_2_ on SLG and BLG, respectively. The relevant quantity here is the work function change between SLG and BLG on SiC, such that the higher work function of BLG pushes the WS_2_ VBM closer to *E*_*F*_^35^, as observed in our data. The difference in Schottky barrier height between SLG and BLG areas results in a built-in bias Δ*ϕ* ≈ 0.3 eV that laterally conforms to the SLG/BLG patterns. The magnitude of Δ*ϕ* is similar to the SLG/BLG work function difference of 0.1–0.2 eV in ultra-high vacuum (UHV)^35^. We speculate that the slightly larger shift in our case can be attributed to a difference in dielectric screening between SLG and BLG, which may give rise to an asymmetric renormalization of the WS_2_ quasiparticle gap, effectively causing a variation in *χ* as well^1,7^. The interpretation of the band alignment in terms of a Schottky contact without Fermi level pinning relies on the quasi-freestanding nature of WS_2_ on graphene^10,36^. It is consistent with the absence of hybridization between graphene and WS_2_ bands in any of our spectra, as well as with the sharp VB features at ${\bar{\mathrm{\Gamma }}}$, in contrast to the situation on metal substrates^9,11,24^.

The pattern of the PL signal in Fig. 3 may be interpreted in terms of the Schottky contact-induced band alignment. Superficially, it appears surprising that the exciton PL energy is nearly identical for the two sample regions with different band alignments. This, however, is well understood. A rigid band offset would not be expected to affect the quasiparticle band gap in the material, and even a screening-induced band gap renormalization, would only be expected to have a minor effect on the exciton binding energy^6^. The change in band alignment can be used to explain the strongly increased trion signal in the BLG areas, as indicated in Fig. 5d. The more *n*-doped WS_2_ would have a strongly increased population of electrons in the conduction band, facilitating the formation of negatively charged electron–electron–hole (eeh) trions when the material is excited by light as sketched in Fig. 5d^20,37^. Our nanoARPES measurements suggest that trion formation would be expected in the SLG areas, whereas our PL measurements indicate that it is actually favored in the BLG areas (Fig. 3). This can still be understood in terms of Fig. 5d, combined with the knowledge that the PL maps were acquired under ambient conditions rather than in UHV. Under ambient conditions, the higher reactivity of SLG compared to BLG leads to the adsorption of impurities and a reversal of the work function difference in the SLG/BLG patterns compared to UHV, as explained in more detail in Supplementary Note 5 and Fig. S5^28^. Since the deposited WS_2_ largely tracks the work function of the underlying SLG/BLG^21^, this is accompanied by a reversal of the band alignment. We note that the band offset between the SLG and BLG regions also implies the existence of a 1D interface with lateral band bending in the WS_2_ VBs, but this is not observable in our experiments because the screening length of graphene on SiC is an order of magnitude smaller than our spatial resolution, as discussed in Supplementary Note 6 and Fig. S6.

The sharp 1D interfaces and the laterally varying band positions of WS_2_ between SLG/BLG areas demonstrate the concept of creating nanoscale devices from a single sheet of 2D material, placed on a suitably patterned substrate. Indeed our conclusions are applicable beyond the Schottky contacts studied here and we envision that similar properties can be induced on patterned insulating materials based on oxides or hexagonal boron nitride^20,38,39^. Particularly intriguing is the complex interplay between electronic and optical properties, that not only allows the confinement of electronic states but also that of more complex objects—such as trions—on the nanoscale, opening a promising avenue for engineering 2D devices.

## Methods

### Growth of WS_2_/graphene/SiC heterostructures

Graphene was synthesized on a semi-insulating (0001) 6H-SiC substrate etched in H_2_ at 200 mbar, during a temperature ramp from room temperature to 1580 °C to remove polishing damage. Graphene growth was carried out at 1580 °C for 25 min in Ar gas, at 100 mbar, and used as a substrate for subsequent WS_2_ growth. WS_2_ islands were synthesized on graphene/SiC at 900 °C by ambient pressure chemical vapor deposition. During the synthesis process, sulfur powders were heated up to 250 °C to generate sulfur vapor. Ar gas flow was used for carrying the sulfur vapor to react with WO_3_ powder.

### Scanning Kelvin probe microscopy

SKPM experiments were carried out in ambient conditions, using a Bruker Icon AFM and Bruker highly doped Si probes (PFQNE-AL) with a force constant ≈0.9 N/m and resonant frequency *f*_0_ of 300 kHz. Double-pass frequency-modulated SKPM (FM-SKPM) has been used in all measurements, with topography acquired first and the surface potential recorded in a second pass. An AC voltage with a lower frequency (*f*_mod_ = 3 kHz) than that of the resonant frequency of the cantilever was applied to the tip, inducing a frequency shift. The feedback loop of FM-KPFM monitored the side modes, *f*_0_ ± *f*_mod_, and compensated the mode frequency by applying an offset DC voltage, equal to the contact potential difference, which was recorded to obtain the surface potential map. The FM-SKPM experiments in Supplementary Note 5 were carried out in ambient air and vacuum (pressure of 1 × 10^−6^ mbar) as described above using an NT-MDT NTEGRA Aura system.

### Photoluminescence mapping

PL spectroscopy mapping was carried out under ambient conditions using a Renishaw inVia confocal microscope-based system with a 532 nm laser line as the excitation wavelength (2.33 eV excitation energy). The laser beam was focused through a 100× microscope objective, with the PL signal recorded in back-scattering geometry, using integration time of 0.1 s/pixel and a lateral spacing of 0.3 μm to acquire the PL intensity maps.

### nanoARPES

Samples were transferred in air to the nanoARPES end-station at beamline I05 at Diamond Light Source, UK. Prior to measurements the samples were annealed up to 450 °C and kept under UHV conditions (pressure better than 10^−10^ mbar) for the entire experiment. Synchrotron light with a photon energy of 95 eV was focused using a Fresnel zone plate followed by an order sorting aperture placed 8 and 4 mm from the sample, respectively. The sample was aligned using the characteristic linear dispersion of the underlying graphene substrate as described in Supplementary Note 1. The standard scanning mode involved collecting photoemission spectra with a Scienta Omicron DA30 hemispherical analyzer by rastering the sample position with respect to the focused synchrotron beam in steps of 250 nm using SmarAct piezo stages. Areas with WS_2_ islands were found using coarse scan modes with larger step sizes as described in Supplementary Note 2. We measured multiple fast maps of the same area on the sample sequentially, and these were subsequently aligned and added together in order to remove possible intensity variations from lateral drifts. The total data acquisition times were typically on the order of 8 h for the scans presented here. The energy- and angular-resolution were set to 30 meV and 0.2°, respectively. The spatial resolution was determined to be (500 ± 100) nm using a sharp feature in the sample as described in Supplementary Note 7 and Fig. S7. The experiments were carried out with the sample at room temperature.

## Supplementary Information

Supplementary Information

## Data Availability

All data presented in this study are available from the corresponding authors upon reasonable request.
