# Supplementary material for: Nanoscale mapping of quasiparticle band alignment
Source: Nat Commun. 2019 Jul 23;10:3283. doi: 10.1038/s41467-019-11253-2 (PMC6650412; doi:10.1038/s41467-019-11253-2)
Supplement: Supplementary file 1 — Supplementary Information [file 41467_2019_11253_MOESM1_ESM.pdf]

Supporting Information: Nanoscale mapping of quasiparticle band alignment

Søren Ulstrup *et al.*

Supplementary Note 1: Nanoscale photoemission from graphene/SiC substrate and sample alignment

We demonstrate that single-layer graphene (SLG) can be spatially distinguished from bilayer graphene (BLG) in a region of the sample devoid of WS_2 islands. Figs. S1(a-b) present spectra from SLG and BLG areas, revealing the expected splitting of the valence band (VB) in the BLG case [1]. The (E, k)-cuts are obtained from a 10 μm line scan of the sample measured in steps of 250 nm. The spatial dependence of the photoemission intensity is represented in Fig. S1(c) by momentum distribution curve (MDC) cuts at a binding energy of 1 eV (see full line labeled MDC cut in Figs. S1(a-b)), as a function of sample position x along the real space line. The presence of BLG is here seen by the appearance of the additional bands marked by arrows. BLG appears in stripes that are up to 1 μm wide, consistent with the scanning Kelvin probe microscopy (SKPM) data in Fig. 1(b) of the main paper.

Two electron pockets are seen in the narrow Fermi surface map along the $\bar{\mathrm{K}}$-$\bar{\mathrm{M}}$-$\bar{\mathrm{K}}'$ high symmetry direction in Fig. S1(d). These correspond to the n-type doped Dirac cones of graphene, which were used to orient the Brillouin zone (BZ) with very high precision. The data in Figs. S1(a-c) were obtained along the red dashed line, which represents the slit direction of our detector. The WS_2 data in the main paper were acquired along the blue dashed line, which was achieved by rotating the polar angle of the detector without changing the orientation of the sample. We outline the edge of the WS_2 BZ via an orange circle since the islands are found to have many different orientations. The islands in Fig. 1 of the main paper were selected for detailed analysis because of the apparent 0° and 30° orientations with respect to graphene, thereby providing access to the $\bar{\Gamma}$-$\bar{\mathrm{M}}$ and $\bar{\Gamma}$-$\bar{\mathrm{K}}$ high symmetry directions of WS_2. This alignment procedure ensures that all spectra cut exactly through the $\bar{\Gamma}$ point, which is essential for determining the energy splittings of the WS_2 VB around $\bar{\Gamma}$.

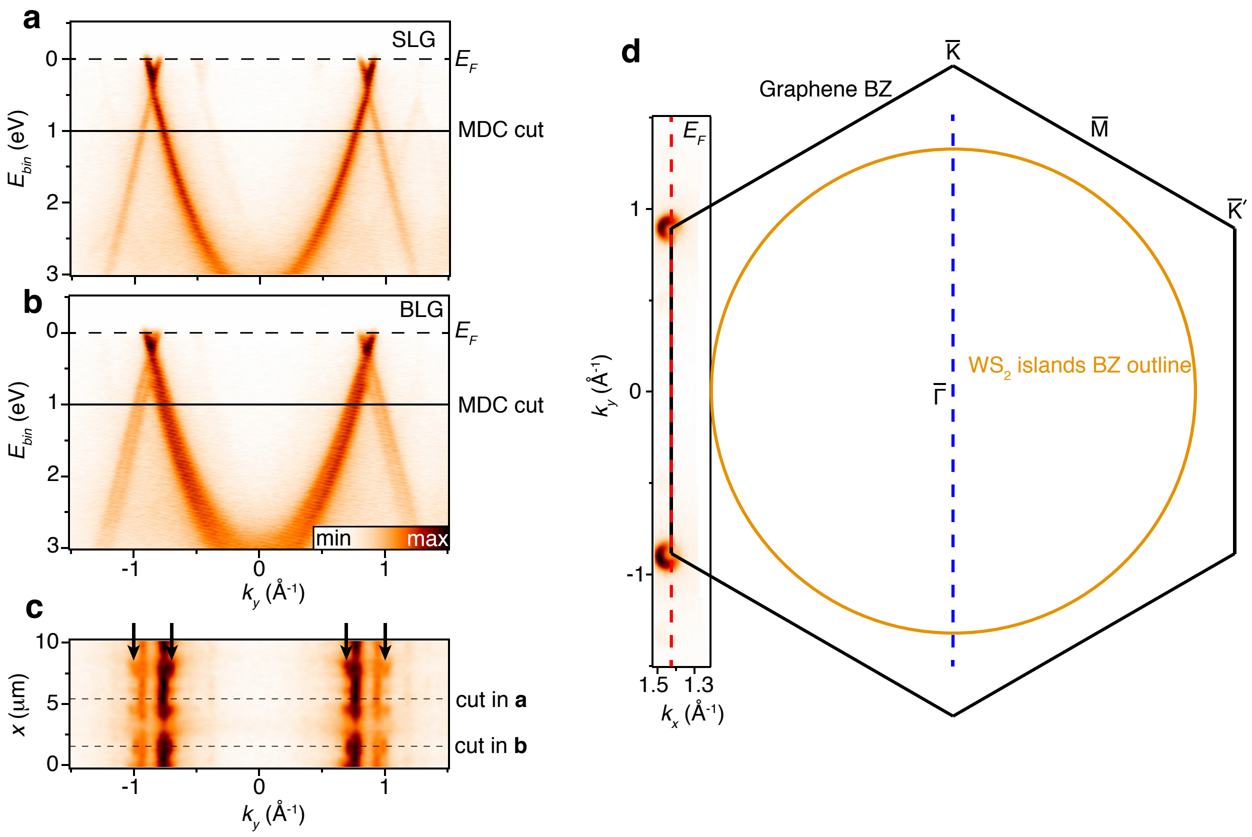

FIG. S1. **Nanoscale electronic structure of graphene/SiC substrate. a-b,** Dispersion of **a** SLG and **b** BLG. **c,** MDCs taken at a binding energy of 1 eV (see full lines in **a-b**) plotted along a line in real space (sample position given by x) in an area without WS_2 islands. Bands associated exclusively with BLG have been marked by arrows. The horizontal dashed lines mark the x-positions where the dispersions in **a-b** have been obtained. **d,** Fermi surface scan around the edge of the graphene BZ (black hexagon). The dispersions in **a-b** were obtained along the red dashed line. The WS_2 VB measurements in the main paper were acquired along the blue dashed line. The measurement was used to reference the BZs of WS_2 islands (edge outlined by orange circle) to the BZ of the graphene substrate.

Supplementary Note 2: Coarse map of WS_2 islands on graphene

The detailed (E, k, x, y)-intensity map around the edges of two WS_2 islands presented in Fig. 1 of the main paper was selected on the basis of a coarse overview map. This is obtained by scanning a region of 30×30 μm^2 in steps of 500 nm. Fig. S2(a) presents a few-layer (FL) WS_2 dispersion from the interior part of a WS_2 island. An (x, y)-map is then composed from the intensity in the region of k-space marked by a blue box in Fig. S2(a), which contains the WS_2 local VB maximum (VBM) at $\bar{\Gamma}$. The resulting map in Fig. S2(b) reveals the location of all WS_2 islands due to the strong VBM contrast, and it makes it possible to set up a fine scan near an island edge, where SL WS_2 is usually found, as in Fig. 1 of the main paper.

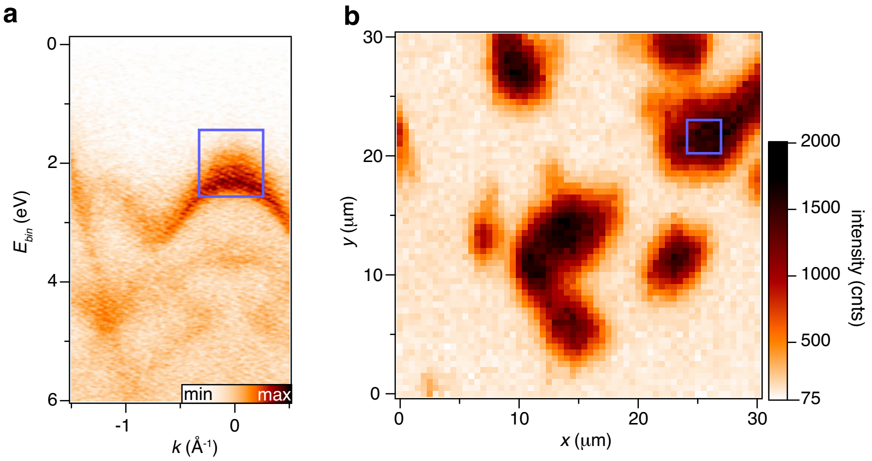

FIG. S2. **Overview photoemission map of the sample. a,** Spectrum from interior WS_2 island, which is associated with FL WS_2. **b,** (x, y)-intensity map integrated within the (E, k)-region marked by a blue box in **a**. The blue box in **b** marks the area and bin-range used to obtain the dispersion in **a**.

Supplementary Note 3: Intensity contribution from few-layer WS_2 islands

The maps in Fig. 1 of the main paper, which have been reproduced in Figs. S3(a-c), contain some pixels of high intensity that do not adhere systematically to the location of SLG and BLG patches. We plot additional spectra around $\bar{\Gamma}$ from the pixels labeled (d)-(h) in Figs. S3(a-c) in the panels with the same labels in Figs. S3(d)-(h) in order to investigate where this intensity is coming from. The blue, green and magenta boxes in Figs. S3(d)-(h) cover the same region of k-space as the similarly colored boxes in Figs. 1(c-f) of the main paper. The intensity is integrated to produce the (x, y)-maps in Figs. S3(a-c).

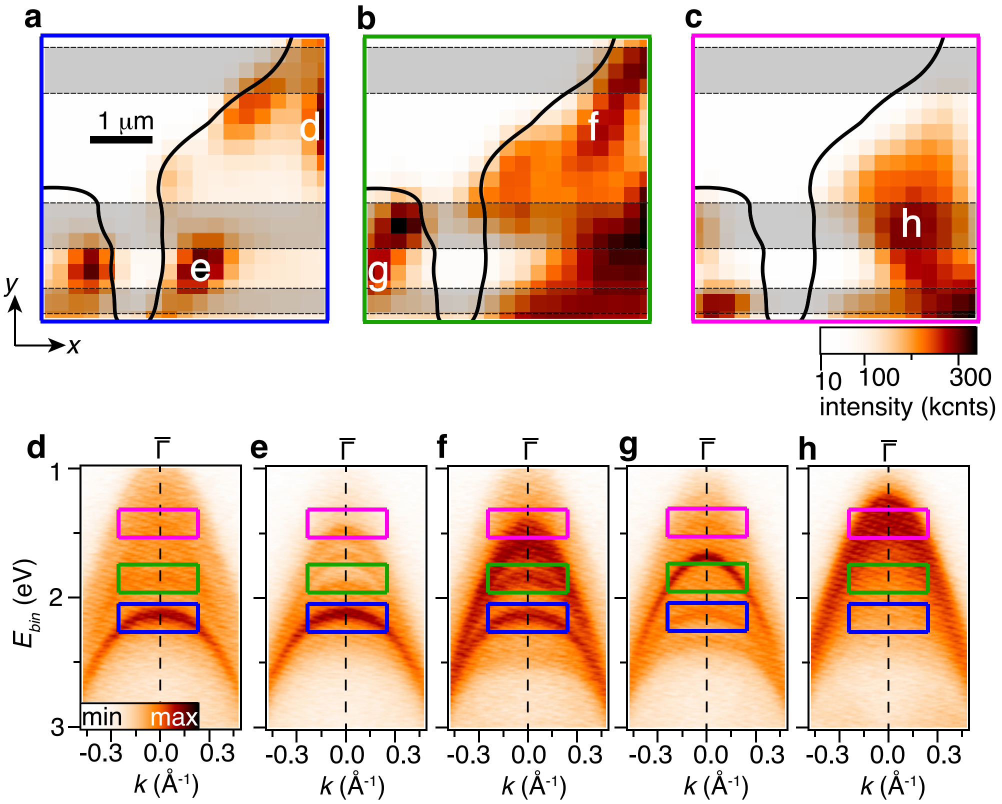

FIG. S3. **Photoemission intensity from FL WS_2. a-c,** Intensity maps integrated within the regions of k-space marked by correspondingly colored boxes in **d-h**. The maps are identical to those in Figs. 1(g-i) of the main paper. **d-h,** Spectra from the areas labeled with the same letter in **a-c** as the panel labels.

The spectra from WS_2 on SLG in Figs. S3(d-e) exhibit an intense VB state at a binding energy of 2.1 eV (see blue box), which we associate with SL WS_2 as explained in the main paper. Additional broad intensity and multiple splittings are observed towards lower binding

energies (overlapping with both green and magenta boxes). The WS_2 on SLG spectra in panels (f-g) exhibit similar bands with multiple splittings but in these cases the intensities are higher, which is giving rise to additional intense pixels in the SLG areas of the map in Fig. S3(b). Since this map is produced from the green box we would expect to see mainly the spatial distribution of the state at $\bar{\Gamma}$ shifted 300 meV towards lower binding energies, which should occur only on BLG areas as discussed in the main paper. However, the multiple splittings appear consistent with FL WS_2 islands because the states around $\bar{\Gamma}$ derive from out-of-plane orbitals that are extremely sensitive to inter-layer interactions [2]. The significant local variations of contrasts from the split bands track the highly heterogeneous coverage and thickness composition of the FL islands that we also observe in the SKPM data in Fig. 1(b) of the main paper. Within 2 μm of the edge of the WS_2 island all the spectra resemble the dispersion in Fig. S3(h), where the VBM is situated at 1.2 eV and is characterized by a broad distribution of intensity without discrete splittings. These observations point towards a strong bulk-like character of the WS_2, already within 2 μm.

Our nanoARPES measurements and analysis in Fig. 1 of the main paper and Fig. S3 demonstrate that nanoARPES is an essential tool to disentangle heterogeneous samples using electronic structure contrasts. Here, we have been able to separate FL WS_2 islands from SL WS_2 areas on SLG and BLG patches using distinct features in the electronic structures of all the material combinations.

Supplementary Note 4: WS_2 electronic structure around $\bar{\Gamma}$ in a triangular island

The key findings of Fig. 1 in the main paper are reproducible across the sample surface. As an example, we show nanoARPES measurements from an isolated WS_2 triangular island in Fig. S4. The analysis carried out in connection with Fig. 1 of the main paper is repeated here in order to test whether similar electronic structure changes occur between SLG and BLG supported regions of a small WS_2 island. The spectrum in Fig. S4(a) is obtained from an edge region of the triangle on SLG. Here, only a single VB state is observed around a binding energy of 2.1 eV (see blue box) as in Fig. 1(c) of the main paper. Fig. S4(b) presents a spectrum from an edge region of the triangle that coincides with a BLG stripe.

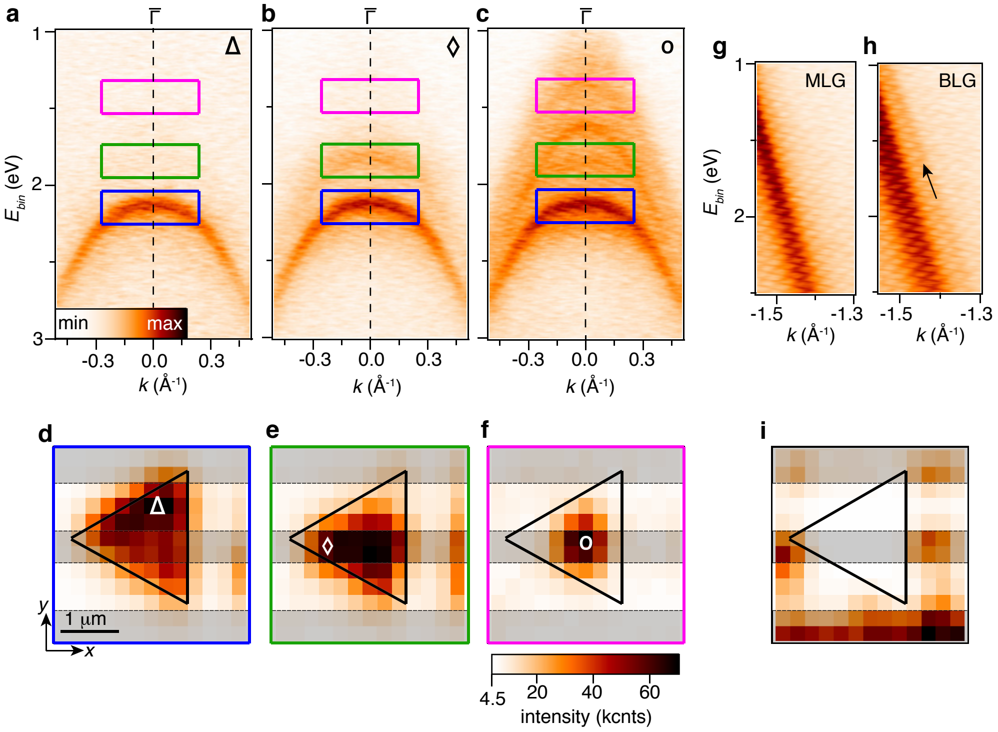

FIG. S4. **Nanoscale photoemission from an isolated WS_2 triangle. a-c,** Spectra around $\bar{\Gamma}$ obtained near the WS_2 triangle edge **a** on SLG and **b** on BLG, as well as **c** in the center of the triangle. **d-f,** Intensity maps integrated within the regions marked by correspondingly colored boxes in **a-c**. A black outline marks the triangular island. The symbols **d** "△", **e** "◇" and **f** "○" mark the coordinates where the spectra with the same symbol labels in **a-c** were acquired. **g-h,** Spectra from **g** SLG and **h** BLG patches. **i,** Map of the intensity from the BLG band marked by an arrow in **h**. The grey-shaded boxes in **d-f** and **i** mark the position of the BLG stripes determined from the map in **i**.

Intensity from a band shifted 0.3 eV to lower binding energy appears (see green box), similar to Fig. 1(e) of the main paper. A spectrum from the center of the triangle contains a very broad distribution of intensity as shown in Fig. S4(c) (see magenta box). A set of (x, y)-intensity maps from these characteristic features can be seen in Figs. S4(d-f). They have been obtained by integrating the intensity in the (E, k)-regions outlined by correspondingly coloured boxes in Figs. S4(a-c). The locations of underlying BLG stripes marked by grey-shaded boxes have been determined by integrating the intensity following the BLG band (compare Figs. S4(g-h) in order to see the BLG band marked by an arrow in panel (h)) and mapping it as in Fig. S4(i). A BLG stripe is seen to cut through the center of the triangle. Due to the narrow width of the BLG stripes, the photoemission intensity from the BLG region in Fig. S4(b) represents an average of the WS_2 VB on BLG and on the surrounding SLG areas. Pixels with high intensity from the high binding energy VB state concentrate on SLG areas (see Fig. S4(d)) while pixels of high intensity from the band shifted 0.3 eV to lower binding energies concentrate on the central BLG stripe in Fig. S4(e). The broad features in Fig. S4(c) give rise to higher intensity at the center of the triangle in Fig. S4(f). We believe these originate from FL structures that tend to grow around the central nucleation site, consistent with observations made by SKPM and photoluminescence. The behavior of the intensity discussed here is qualitatively in line with the region studied in Fig. 1 of the main paper.

Supplementary Note 5: Work function change in ambient and vacuum

Surface potential images in Figs. S5(a)-(b) acquired using SKPM illustrate the effect of vacuum and ambient environment on the work functions of SLG, BLG and WS_2 islands on SLG and BLG. In ambient (Fig. S5(a)), the surface potential image shows BLG as bright parallel stripes at the edge of SiC terraces superimposed on a dark background of SLG. The BLG stripes are also visible in areas with WS_2 on graphene. Contrast inversion of the surface potential relative to ambient conditions is observed in vacuum (Fig. S5(b)), where

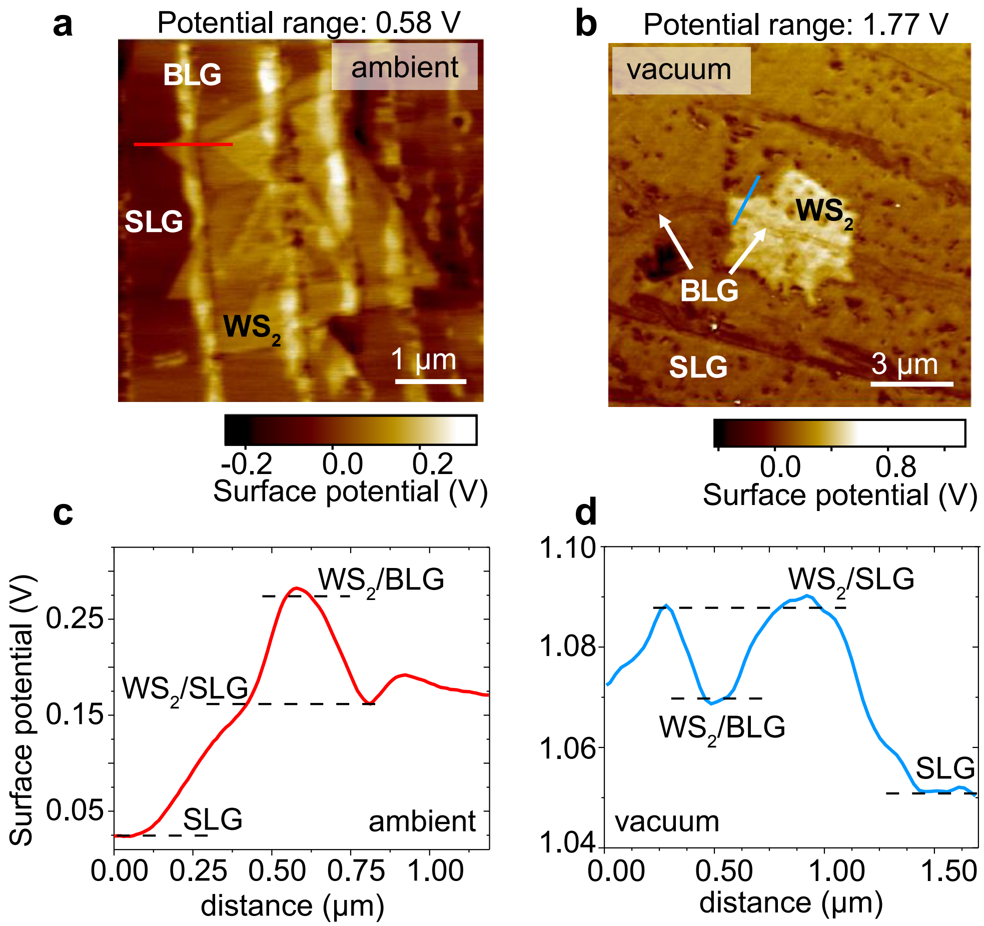

FIG. S5. **SKPM of WS_2/graphene heterostructures in ambient and vacuum. a-b,** Surface potential maps in **a** ambient and **b** vacuum conditions. **c-d,** Corresponding surface potential line profiles taken along the red and blue lines indicated in **a** and **b**, respectively. The bright contrast associated with BLG in **a** corresponds to the WS_2/BLG peak in **c**, and the dark contrast for BLG in **b** to the WS_2/BLG dip in **d**.

BLG displays a darker contrast than SLG. The swap in contrast is due to the desorption of environmental adsorbates, such as water and other electron withdrawing species present in the ambient, as discussed in detail in our previous study [3]. The work function values for SLG and BLG in vacuum decrease with respect to ambient conditions, as would be expected from the removal of *p*-type dopants; however, a greater change in work function is observed for SLG than for BLG on the transition from ambient to vacuum. The WS_2 on BLG areas exhibit similar contrast inversion as bare BLG, which is highlighted by the line profile analysis performed on the images obtained in ambient and vacuum conditions in Figs. S5(c)-(d), respectively.

Supplementary Note 6: SL WS_2 band structure around the SLG/BLG 1D interface

A finite bias of ≈ 300 meV between SL WS_2 on SLG and on BLG areas implies the existence of a lateral depletion region and band bending in the SLG and BLG sides. We here investigate whether such lateral band bending can be resolved using both the isolated triangular island from Fig. S4 and an island from Fig. S3 (Fig. 1 of the main paper).

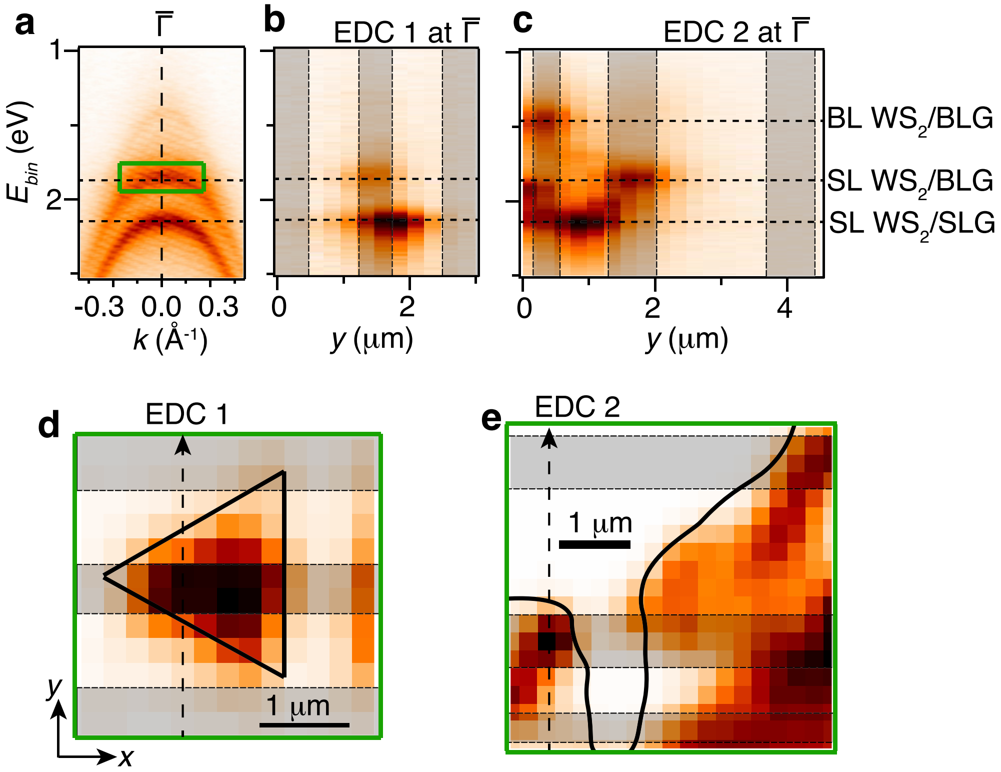

FIG. S6. **Spatial dependence of WS_2 VB EDCs. a,** Laterally averaged spectrum of SL WS_2 on SLG and BLG areas. The VB states from the two areas are marked by horizontal dashed lines. **b-c,** EDCs at $\bar{\Gamma}$ (see vertical dashed line in **a**) stacked as a function of position across WS_2 islands and SLG/BLG interfaces. The BLG areas are marked by grey-shaded boxes. Horizontal dashed lines mark distinct SL and BL WS_2 VB splittings as noted in panel **c**. **d-e,** Intensity maps integrated within the green box in **a**. The vertical dashed arrows in **d-e** mark the locations and directions of the EDC stacks in **b-c**, respectively.

We focus on the SL WS_2 bands from the SLG and BLG areas, which are both seen via horizontal dashed lines in a laterally averaged spectrum in Fig. S6(a). We then plot energy distribution curves (EDCs) at $\bar{\Gamma}$ and stack them as a function of sample position as shown

in Figs. S6(b)-(c). The (x, y)-maps in Fig. S6(d)-(e) were obtained using the integrated intensity in the green box in Fig. S6(a). The intense pixels mainly track the band in the green box on the BLG areas, as explained previously. The dashed vertical arrows in panels (d-e) mark the location and direction of the EDC stacks in panels (b-c) and were selected to cut through mainly SL WS_2 on both SLG and BLG patches.

The EDC stacks in Figs. S6(b-c) reaffirm that the 300 meV shifted WS_2 VB coincides with BLG stripes (see grey-shaded boxes). However, we find in both panels that the two VBs are separated by abrupt binding energy offsets. This implies that we can not resolve the lateral band structure changes around the SLG/BLG interfaces. Indeed, capacitance spectroscopy measurements of n-doped graphene on SiC provide values for the screening length of 66 nm [4], which is an order of magnitude lower than our spatial resolution and significantly below our scan step size.

Supplementary Note 7: Spatial resolution

We estimate the nanoARPES spatial resolution by taking a photoemission intensity profile through a WS_2 triangle, as shown in the insert of Fig. S7. We assume that the edge is sharp compared to our resolution and fit a step function convoluted by a Gaussian. We take the full-width at half maximum (FWHM) of the Gaussian, Δx, as a measure of the spatial resolution in our experiment. We obtain $\Delta x = (500 \pm 100)$ nm as shown in Fig. S7. This value matches measurements using a knife edge profile, yielding a value of 600 nm at the same photon energy. The slight difference can be attributed to larger travel range used for the knife edge profile (at least 5 μm).

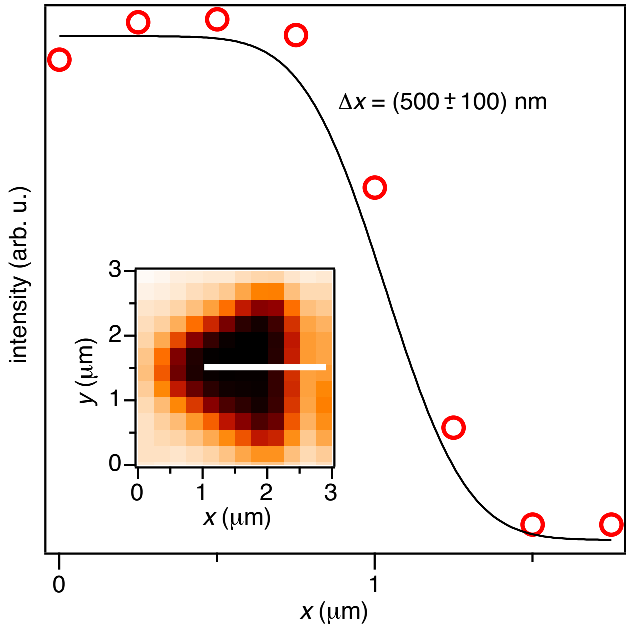

FIG. S7. **Estimate of spatial resolution.** Photoemission intensity profile through the edge of a WS_2 triangle (open circles). The profile is fitted (curve) with a step function broadened by a Gaussian with the given FWHM (Δx) value.

SUPPLEMENTARY REFERENCES

[1] Taisuke Ohta, Aaron Bostwick, J. L. McChesney, Thomas Seyller, Karsten Horn, and Eli Rotenberg, "Interlayer interaction and electronic screening in multilayer graphene investigated with angle-resolved photoemission spectroscopy," Physical Review Letters **98**, 206802 (2007).

[2] Hualing Zeng, Gui-Bin Liu, Junfeng Dai, Yajun Yan, Bairen Zhu, Ruicong He, Lu Xie, Shijie

Xu, Xianhui Chen, Wang Yao, and Xiaodong Cui, "Optical signature of symmetry variations and spin-valley coupling in atomically thin tungsten dichalcogenides," Scientific Reports **3**, 1608 (2013).

[3] Cristina E. Giusca, Vishal Panchal, Martin Munz, Virginia D. Wheeler, Luke O. Nyakiti, Rachael L. Myers-Ward, D. Kurt Gaskill, and Olga Kazakova, "Water affinity to epitaxial graphene: The impact of layer thickness," Advanced Materials Interfaces **2**, 1500252 (2015).

[4] S. Sonde, C. Vecchio, F. Giannazzo, R. Yakimova, V. Raineri, and E. Rimini, "Effect of graphene/4H-SiC(0001) interface on electrostatic properties in graphene," Physica E: Low-dimensional Systems and Nanostructures **44**, 993 – 996 (2012).
